# Design and Validation of an Observational System for Penalty Kick Analysis in Football (OSPAF)

**DOI:** 10.3389/fpsyg.2021.661179

**Published:** 2021-05-28

**Authors:** Guilherme de Sousa Pinheiro, Vitor Bertoli Nascimento, Matt Dicks, Varley Teoldo Costa, Martin Lames

**Affiliations:** ^1^Chair for Performance Analysis and Sport Informatics, Department of Sports and Health Sciences, Technical University of Munich, Munich, Germany; ^2^Motor control and Biomechanics, Department of Sports, State University of Londrina, Londrina, Brazil; ^3^School of Sports, Health and Exercise Sciences, University of Portsmouth, Portsmouth, United Kingdom; ^4^UFMG Soccer Science Center, Department of Sports, Federal University of Minas Gerais, Belo Horizonte, Brazil

**Keywords:** performance analysis, observational methodology, content validity, Aiken’s V, elite football

## Abstract

The analysis of penalty kick has played an important role in performance analysis. The study aims are to get formal feedback on the relevance of variables for penalty kick analysis, to design and validate an observational system; and to assess experts’ opinion on the optimum video footage in penalty kick analysis. A structured development process was adopted for content validity, reliability and agreement on video usage. All observational variables included in OSPAF showed Aiken’s *V* values above the cut-off (for 5-scale *V*> 0.64; for 2-scale = *V* > 0.75; *p* < 0.05). Cohen’s Kappa resulted in mean intra- and inter-rater reliability values of 0.90 and 0.86, respectively. It is recommended to combine at least three different viewing angles (*V* = 0.90; *p* = 0.006) with standardization of video quality (*V* = 0.95; *p* = 0.006). Changing the viewing angles may influence the observer perception (*V* = 0.86; *p* = 0.006). The aerial and pitch-level viewing angle behind the penalty taker and pitch-level viewing angle behind the goalkeeper were indicated as most appropriate for observational analysis (*V* = 0.97; *p* = 0.01). The OSPAF met all requirements of instrument validation. It may be recommended as basis of future observational systems on penalty kicks.

## Introduction

Penalty kicks play a decisive role in the outcome of a match in competitive football (Makaruk et al., 2020; Paterson et al., 2020). UEFA introduced penalty shoot-outs to major tournaments in 1976 (FIFA followed in 1978) as a means of deciding matches in the knockout phase of major tournaments when the score is a draw at the end of the match. Many important competitions were decided by penalties, for example, in the 2018 World Cup, 25% of the matches in the knockout stages were decided by penalty shoot-outs. In the big five European leagues, the average number of penalties per game since the 2017/18 season is 0.31 (Instat, 2020). It has been estimated that about 30–40% of the goals are scored from set plays (i.e., penalty kicks, free kicks, corners, and throw-ins) (Fariña et al., 2013; Sarmento et al., 2018). Among these set plays, the penalty is the situation with the highest chance of scoring a goal. The penalty situation can be considered as the peak of high-pressure performance in elite football (Wood et al., 2015; Brinkschulte et al., 2020). Notably, researchers have shown a significant interest in uncovering factors that affect success in the penalty kick (Paterson et al., 2020).

To further improve the performance of football players, a large amount of data is produced in professional leagues that provide many options to analyze games and identify critical factors for success (Lepschy et al., 2020). Penalty kicks have been mainly analyzed in two contexts: First, in a laboratory or other non-game controlled settings (video-simulation and in-situ experimental conditions), aiming at the analysis of perceptual-motor and cognitive aspects of performance (e.g., Dicks et al., 2010; Lopes et al., 2012; Weigelt and Memmert, 2012; Navarro et al., 2013); and second, in real match situations, enabling the identification of prominent factors that affect both players’ performances and the penalty kick outcome using mainly observational methods (e.g., Chiappori et al., 2002; Jordet et al., 2007; White and O’Donoghue, 2013; Horn et al., 2021). While in the first context, a common theoretically motivated focus has been developed to enhance the representative design of methods used to examine the expertise of penalty takers and goalkeepers (Dicks et al., 2009), in the second, researchers have attempted to improve data collection procedures based on game video analysis (Almeida et al., 2016).

From a behavioral perspective, the penalty outcome depends, above all, on the emerging results of the “penalty taker—goalkeeper” dyadic interaction (Lopes et al., 2012; Almeida et al., 2016). Reviews on performance analysis have suggested that the future of game analysis in football requires the building of observational instruments and analytical procedures that integrate the study of criteria related to the interactions between opponents (Mackenzie and Cushion, 2013; Sarmento et al., 2014). Notational analyses are scientific procedures that reveal the occurrence of perceivable behaviors, allowing them to be formally recorded and quantified (Anguera et al., 2001). To support top-level teams two purposes of game observation are predominant: preparation against a future opponent and the optimization of training (Lames and Hansen, 2001; Lames and McGarry, 2007). The analysis, therefore, aims to describe the participants’ behavior during real competitive scenarios, concerning different tactical, technical, and performance aspects (Vázquez-Diz et al., 2019).

Although observational studies are frequently used and their utility in different contexts has been widely proven, there are concerns regarding the information related to validity and reliability concerning the processes of systematic observation (Chacón-Moscoso et al., 2018). As a prerequisite for any performance analysis research that uses a novel system or instrument, the repeatability and accuracy of this new tool and the validity of performance indicators it provides should be tested before collecting and analyzing players and teams’ performances (O’Donoghue, 2014; Gong et al., 2019).

Validity and reliability are important criteria for any scientific measurement. Validity is generally referred to as the ability of a measurement tool to reflect what it is designed to measure, and usually, for performance analysis instruments, it can be determined through expert coaches’ opinions in each sports category (O’Donoghue, 2009; AERA et al., 2014). Reliability is the consistency of a measure and is a part of the evidence of validity (Sullivan, 2011; Heale and Twycross, 2015). It refers to the reproducibility of values of a test, assay, or other measurements in repeated trials by the same individuals (intra-observer reliability) and repeatability over different observers (inter-observer reliability) (O’Donoghue, 2009; Gong et al., 2019). Because the human observer is the measurement instrument in observation, it is highly recommended to establish content validity and reliability of notational systems and observational instruments to reduce the error caused by human subjectivity (Lames, 1994; O’Donoghue, 2009; Taherdoost, 2016; Cobb et al., 2018).

Previous research has used a development process to evidence content validity of an observational instrument (Brewer and Jones, 2002; Fernandes et al., 2019). This process includes several sub-stages, such as literature review, instrument development, the establishment of content validity with experts, observer training, pilot study, inter-observer reliability, and intra-observer reliability assessment. This suggested development process has guided the present study.

Several studies have investigated the penalty kick strategies in football (e.g., Savelsbergh et al., 2005; van der Kamp, 2006; Bowtell et al., 2009; Vega Marcos et al., 2010; Zuo et al., 2010; Lopes et al., 2012; Timmis et al., 2014; Furley et al., 2017; Sarmento et al., 2018; Makaruk et al., 2020), however, only a few studies proposed instruments to examine this set piece. For example, Comas et al. (2018) created a system based on an observational methodology with to analyze the direction of the ball at a penalty kick in football. These authors pointed out a relationship between the spatial position of the support foot and the opposite arm to the shooting foot with the direction of the ball on the penalty kick, both for right-footed and left-footed players. Noël et al. (2015) developed a method for identifying the determinants of penalty kick strategies in a controlled simulated situation, before evaluating penalty kick performances using video footage from competitive matches. They included 12 variables in this observational system. A logistic regression model identified three variables (attention to the goalkeeper, run-up fluency, and kicking technique) that in combination could predict kick strategy in 92% of the penalties. However, one possible limitation is that the penalty takers followed a script denoting whether they use a keeper independent or dependent strategy and therefore the design did not reflect the unfolding of an interaction between the two players. In real competitions, penalty kicks are an interaction process, and the observable performance is rather the emergent result of this interaction process than the display of skills and abilities of the two parties (Lames, 2006; Lames and McGarry, 2007). Future research needs to address how these factors affect the validity of the instrument.

Performance indicators play a key role in contemporary sports analytics (Sampaio and Leite, 2013). In training and coaching, these metrics play an important role mostly as starting point for a more in-depth qualitative game analysis (Lames and Hansen, 2001; Carling et al., 2014). There are several studies analyzing penalties in field settings (Dalton et al., 2015; Wood et al., 2015; Brinkschulte et al., 2020; Higueras-Herbada et al., 2020; Wunderlich et al., 2020; Horn et al., 2021). Most of them do not aim at giving a detailed description of the actions of the shooter and goalkeeper, but focus more on statistical results (e.g., quotes for scoring and saving penalties of different kinds). Despite the extensive coverage in the literature of penalty kicks in elite football and methods developed for the analysis of penalty taker actions (Noël et al., 2015), there is no scientific consensus concerning observational variables to use for the analysis of both goalkeeper and penalty taker actions. The development of this instrument enables the collection of data using systematic observation. Differentiating between penalty kick patterns would be of both scientific and applied interest. This would allow researchers to identify determinants of successful kicks (e.g., patterns of gaze) especially under high pressure, as well as facilitating future comparisons between investigations on this topic. Also, practitioners in professional football could distinguish penalty kick strategies and so inform coaching, training, and scouting. OSPAF may serve as a standard tool for observational investigations of penalties in football to make the results from different studies more comparable. This would allow for replications of studies to track for example long-time trends and also for comparisons between different settings (e.g., countries, leagues, age groups, gender). Therefore, a methodological design containing three studies (pilot study, main study, and video requirements study) was carried out. The aim of (1) the pilot study was to get formal feedback on variables for penalty kick analysis suggested by professionals in the area; (2) the main study aimed at designing and validating an observational system applied to in-match penalty kick analysis; and (3) the video study served to evaluate the influence of the video footage (i.e., viewing angles, number of angles and video quality) on penalty kick analysis through an observational system.

## Materials and Methods

### Pilot Study

Participants in the pilot study were four sports scientists and three high-level football coaches (43.32 ± 15.48 years). The inclusion criteria established for forming part of the panel of sports scientists were: (1) postgraduate master in sports sciences or Ph.D. in sports sciences, (2) to have had at least 3 years experience as a university researcher in sports sciences, (3) experience in performance analysis research (final master’s thesis, doctoral thesis or scientific publication); and for high-level football coaches were: (1) graduate in physical activity and/or sport sciences, (2) have an official license as a football coach, (3) more than 3 years as a football coach in a team of an official competition. They evaluated and provided judgment on the instrument’s variables. All participants provided informed consent after details of the study were communicated in written form before participation in the study. All procedures performed in the study were in strict accordance with the Declaration of Helsinki as well as the ethical standards of the Technical University of Munich.

The pilot study refers to a mini version of the full-scale study, as well as the specific pre-testing of the particular research instrument, here the online questionnaire (Van Teijlingen and Hundley, 2001). The pilot study aimed to was to get formal feedback on variables for penalty kick analysis and to collect observable variables suggested by professionals in the area. A survey, developed in Google Forms, was used to assess the content validity of the proposed observational system (Fernandes et al., 2019). A link for the online survey was emailed to the participants. They were instructed to answer the questionnaire on a computer or notebook, and there was no time limit to answer the questions.

### Main Study

A panel of 20 experts (41.85 ± 13.96 years), from Brazil, England, Germany, Israel, Netherlands, Romania, and Spain, who met the following criteria: (1) Ph.D. in sports sciences, and (2) experience of publishing in penalty kick research was contacted and voluntarily agreed to participate. More detailed characteristics about the experts were collected, such as sports biography and open items on the experts’ general judgment on each criterion. All participants provided informed consent after details of the study were communicated in written form before participation in the study. All procedures performed in the study were in strict accordance with the Declaration of Helsinki as well as the ethical standards of the Technical University of Munich.

The main study aimed to design and validate an observational system applied to in-match penalty kicks analysis and to follow a systematic process to accumulate evidence of content validity and reliability to adequately categorize and record behaviors of both penalty takers and goalkeepers during penalty kicks. The process to achieve content validity for the OSPAF is described below in different stages, adapted from Brewer and Jones (2002) and Fernandes et al. (2019):

#### Content Validity With Experts

The panel of experts answered the survey in web format and the level of concordance among experts for each of the variables proposed in the OSPAF was analyzed. A modified Delphi method was performed (Dalkey and Helmer, 1963; Hasson et al., 2000; Dayé, 2018). For concordance analysis three dimensions were defined (Fitzpatrick, 1983; Fernandes et al., 2019):

• Agreement: the degree of general acceptance of the variables to be included in the observational system. The question in the survey was: How is your level of agreement with the inclusion of the variable for penalty kick analysis in the proposed system? A five-point Likert scale (Strongly disagree, Disagree, Neither disagree nor agree, Agree, Strongly agree) was utilized.
• Univocity: clarity domain of a definition; a binary scale (Yes or No). The question in the survey was: The definition of the variable is clear enough for understanding?
• Adequacy: level of pertinence and importance of criteria. The question in the survey was: What is the level of importance of the variable for the observational system? A different five-point Likert scale (Very low, Low, Medium, High, Very high) was applied (Jamieson, 2004).

#### Inter- and Intra-Observer Reliability

The verification of the reliability of OSPAF was made through the assessment of Cohen’s kappa (κ) between observers (inter-observer agreement) and for the analysis of interpretative stability within one observer (intra-observer agreement). For the inter-observer agreement, apart from the analysis carried out by the main researcher, a second researcher was trained in the analysis of the penalty kicks with OSPAF. After the training period, the two observers independently analyzed 40 randomly selected penalty kicks of the World Cups 2014 and 2018. Regarding the intra-observer agreement, the principal investigator performed the same analysis 4 weeks after the first analysis thus minimizing task familiarity (Robinson and O’Donoghue, 2007), without conducting any type of analysis during this time, thus checking the temporal stability of the analysis (Aranda et al., 2019).

### Video Study

#### Participants and Procedures

The same panel of 20 experts as in the main study participated also in this third study, aiming to evaluate the influence of the viewing angles for penalty kick analysis through an observational system. Using an online questionnaire, 14 penalty kick videos from elite football each from 7 different angles were presented (Supplementary Figure 1). The methodology adopted in the present study is similar to Baranowski and Hecht (2017) (i.e., fifteen-second scenes used as examples, and later on a questionnaire was applied to gather feedback). The videos had a pixel resolution of 1,280 × 720. The experts should indicate which were the best viewing angles for penalty kick analysis. They were instructed to watch the videos on a computer or a notebook.

The choice of angles was adapted from a division of the field into zones proposed by Garganta (1997) and previously used by Moraes et al. (2014). This corresponds to the topographical division of the playing field, and its use ensured the establishment of spatial references for choosing the angles. Experts could watch each penalty kick video as many times as they judged necessary.

Besides, the experts were asked about how many viewing angles were needed for penalty kick analysis; whether changing the viewing angle could influence the observer’s analysis, and whether video quality is a basic prerequisite for standardizing penalty kick analysis using an observational system.

The panel of experts answered the survey in web format and the level of concordance among experts for the following domains were analyzed:

• The number of angles needed for penalty analysis: The question in the survey was: In your opinion, how many video angles are required for the evaluation of a penalty kick in observational studies? A five-point Likert scale (1 video angle, combination of 2 video angles, combination of 3 video angles, combination of 4 video angles, combination of 5 or more video angles) was utilized.
• Influence of changing angles on the observer’s analysis: The question in the survey was: In your opinion, changing the angle presented could influence the evaluation of penalty kicks by an observer? A binary scale (1. Yes or 0. No) was used.
• Pre-requisite of video quality: The question in the survey was: In your opinion, the video quality is a prerequisite for penalty kick analysis in football? A binary scale (1. Yes or 0. No) was used.

### Instrument

For the pilot and main study, a survey with two different versions developed in Google Forms to assess content validity with the experts. For the video study another online survey, containing penalty kick videos (i.e., 2016 Olympics, World Cups between 2010 and 2018, and major European leagues from 2015 to 2020) was utilized. For reliability, the final version of the OSPAF was used after implementation in Lince Plus software (Gabin et al., 2012; Soto et al., 2019). Lince Plus is free software that has been used by many researchers needing a tool to tag behaviors using video recordings, coding behaviors, and data register (Soto et al., 2019). Dimensions and categories of OSPAF were coded and the observations of the two observers were compared using this software. Criteria were entered with the full definition of the variable (i.e., Run up speed), and categories were coded with the initials letters (i.e., Fast = F and Slow = S), as illustrated in the figure below.

### Statistical Analysis

For descriptive analysis, mean and standard deviation were used. Aiken’s *V* was calculated (Aiken, 1985) for content validity of the OSPAF variables and to evaluate the level of agreement of the experts according to the number of angles needed for penalty analysis; the influence of changing angles on the observer’s analysis; and the pre-requisite of video quality. Aiken’s *V* allows for quantifying the relevance of items expressed in Likert scales, according to the opinions of a group of experts. Its values vary between 0 and 1, with 1 indicating a perfect agreement among the judges. Previous studies have used the same coefficient to establish validity in observational instruments (Villarejo et al., 2014; Garcia-Santos and Ibanez, 2016; Fernandes et al., 2019; Ortega-Toro et al., 2019). The *p* level considered for Aiken’s *V* was 0.05 and a 95% confidence interval was used. The score confidence interval was used to provide the expected accuracy of Aiken’s *V* value (Randall et al., 2009). The calculation of Aiken’s *V* is as follows:

$$V\;\frac{\sum s}{n\,\left(c-1\right)}$$

Description: *n* = number of judges; *c* = highest value of Likert scale; *s* = *r* – *l*; *r* = the judgement given by a judge; *l* = lowest value of Likert scale.

For each dimension (agreement, univocity, and adequacy), the criteria for the elimination or acceptance of the items were fixed in advance. The reference table proposed by Aiken (1985) for samples with *n* < 25 was used (number of rating categories: 5; *V* = 0.64, *p* < 0.05; number of rating categories: 2; *V* = 0.75, *p* < 0.05). Consequently, variables with Aiken’s *V* values below cut-off values of 0.64 in agreement or adequacy, or univocity below 0.75, were eliminated.

For intra- and inter-reliability of the OSPAF, Cohen’s kappa was calculated. The interpretation of this coefficient was adopted as follows: κ > 0.8 very good; 0.6 < κ < 0.8 good; 0.4 < κ < 0.6 moderate; 0.2 < κ < 0.4 fair; κ < 0.2 poor (Altman, 1991; O’Donoghue, 2009). For the specific objectives of this study only values κ > 0.8 were considered satisfactory (Lames, 1994).

To identify the best angle for penalty analysis, descriptive statistics were used. Microsoft Excel 2016 was used to calculate the values of Aiken’s *V* and confidence interval; Lince Plus software to record the behaviors (Gabin et al., 2012; Soto et al., 2019). The Statistical Package for the Social Sciences software (IBM Corp. Released 2016. IBM SPSS Statistics for Windows, Version 24.0. Armonk, NY: IBM Corp.) was used to calculate Cohen’s Kappa.

## Results

### Pilot Study

The first version of the new penalty kick analysis system was created, based on the collection of several variables from previous studies (Hughes and Wells, 2002; Timmis et al., 2014, 2018; Noël et al., 2015; Almeida et al., 2016; Furley et al., 2017; Comas et al., 2018). Characteristics were selected that are likely to distinguish the profile of successful or unsuccessful penalty kicks and strategies. Furthermore, contextual factors were included (e.g., location of the match; the result of the match at the time of the penalty kick; penalty kick during the normal time or extra time). This first step enabled the development of the first round of content validity of the proposed observational system, with the variables and their definitions.

Qualitative feedback was also gathered from the professionals about the conduct of the online questionnaire. The questionnaire was indicated by 65% of the experts as being very long and complex. In this way, the design of the questionnaire was adjusted for the main study, by repositioning the descriptions of the variables close to the answer box. The use of an online survey presented no problems and was considered a suitable tool for further expert participation.

All the 27 variables proposed in the pilot study have been pointed out by the experts as relevant for the analysis of penalties in football. Aiken’s *V* for Agreement (*p* < 0.05) ranged from 0.66 to 0.89 (cut-off: 0.64); for Adequacy (*p* < 0.05) from 0.64 to 0.83 (cut-off: 0.64) and for Univocity (*p* < 0.05) from 0.89 to 1.0 (cut-off: 0.75). Moreover, the experts suggested the variables ball speed, match importance, and goalkeeper initial posture, which were added to the observational system.

### Main Study

The results regarding content validity in the main study were obtained by calculating Aikens’ *V* and the 95% confidence interval. Data are shown in Supplementary Table 1. All observable variables of the OSPAF, presented in Supplementary Table 1, showed Aiken values above the cut-off value (*p* < 0.05).

Unlike in the pilot study, the following variables have not achieved the minimum values for 5-scale (*p* < 0.05; *V* < 0.64, *n* = 20) and/or for 2-scale (*p* < 0.05; *V* < 0.75; *n* = 20) to be included in the final version of the OSPAF and were excluded. Supplementary Table 2 shows the excluded variables.

The following Supplementary Table 3 presents the final version of the OSPAF and the operational definitions.

Supplementary Table 4 shows the Kappa values for each of the variables of the observational system for penalty analysis in football (OSPAF). From the 24 items validated through the Aiken coefficient, 19 obtained Kappa values above 0.80 for intra-reliability, and the other 5 items values above 0.75. Regarding the inter-reliability, 17 items presented Kappa values above 0.80, the 7 remaining items had values above 0.70. Thus, Kappa values indicate a high level of reliability of the proposed new penalty kick analysis system.

### Video Analysis Study on Optimum Video Footage

The preferred angles for observational analysis of penalty kicks, indicated by the panel of experts in the present study, are shown in Supplementary Figure 2.

It was presented to the experts 14 penalty videos with 7 different viewing angles. 71.4% of the experts indicated the angle c (Behind the penalty taker aerial view), 18.2% the angle d (Behind the penalty taker pitch view), and 10.4% the angle e (Behind the goalkeeper aerial view). The experts agreed on the following methodological requirements: to analyze the penalty in the game through an observational system it is necessary to combine at least 3 different viewing angles of the same penalty (*V* = 0.90; *p* = 0.006). The change of the viewing angles can influence the analysis of the observer (*V* = 0.86; *p* = 0.006). Moreover, the standardization of video quality is a prerequisite for notational analysis (*V* = 0.95; *p* = 0.006).

## Discussion

Three studies with different aims were conducted to approve an observational system for in-match penalty kick analysis: (1) the pilot study to get formal feedback on variables for penalty kick analysis suggested by professionals in the area; (2) the main study to design and validate the observational system; and (3) the video study to evaluate the influence of the video footage.

The first version of the observational system was created and validated with practitioners. All 27 proposed observational variables were considered relevant. Also, the practitioners suggested the inclusion of the following variables ball speed, match importance, and goalkeeper initial posture. Technical adjustments have been made about the conduct of the online questionnaire to better understand the proposed questions, e.g., repositioning of questions, explanations, and videos. As study 1 was considered a pilot, it provided a point of discussion for further studies in the present research. One of the advantages of conducting a pilot study is that it might give warning about where the main research project could fail, where research protocols may not be followed, or whether proposed methods or instruments are inappropriate or too complicated. They fulfill a range of important functions and can provide valuable insights for other researchers (Van Teijlingen and Hundley, 2001).

Since the study objectives are oriented to the construction of an observation instrument, the results refer to the quality control of the data, focused on the Aiken’s *V* values, intra and inter-observer agreement. The Aiken’s *V* value, measured in the dimensions Agreement, Univocity and Adequacy, indicated that content validity was achieved (For 5-scale: *p* < 0.05 and *V*> 0.64; for 2-scale: *p* < 0.05 and *V* > 0.75). The methodological rigor adopted and discussed in the study provides sports scientists, coaches, and professionals involved in football an instrument capable of assessing important indicators in a penalty kick in elite football. Although some variables were indicated as valid for penalty kick analysis in the pilot study (i.e., run-up type, run up length, swing behavior of the kicking leg, position of the arm opposite the kicking leg, preparation time, distraction by the goalkeeper, presence of advertisement behind the goal), in the main study they did not reach the minimum values to be included in the final version of the observational system. These findings might be partially explained by the fact that the panel of experts participating in the main study was larger (*n* = 20) than in the pilot study (*n* = 7). Besides, the panel in the main study had substantial scientific research experience in performance analysis. This may lead to a more detailed analysis of the research design.

The kick strategy of the penalty taker is commonly distinguished as being either goalkeeper-dependent or goalkeeper-independent (Kuhn, 1988). Adopting the “goalkeeper-independent” strategy the penalty taker has a pre-established plan about the direction of the kick and ignores any action of the goalkeeper during the preparatory period (run-up). Alternatively, using the “goalkeeper-dependent” strategy the kicker intends to take advantage of the goalkeeper’s anticipatory action. During the run-up, the penalty taker tries to obtain information from the actions of the goalkeeper in an attempt to anticipate which side the goalkeeper will dive. The analysis of the kick strategies has been investigated about numerous factors, such as spatiotemporal (e.g., run-up, ball speed; Kuhn, 1988; Noël et al., 2015), foot orientation (e.g., the direction of the supporting foot; Li et al., 2015), perceptual (e.g., visual search behaviors; Noël et al., 2015), individual (e.g., footedness; Instat, 2020), psychological (e.g., team status, kick importance). Although all variables included in OSPAF presented Aiken’s *V* value above the cut-off threshold, few variables showed lower values (i.e., number of steps, foot used to kick, and run-up approach angle). This low rating contrasts previous findings which indicate that the foot used to kick can reveal cues for penalty shooting analyses. The dominant foot height and the dominant foot angle is also correlated with the height of the shooting in a penalty kick (Higueras-Herbada et al., 2020). Other authors have indicated that the footedness may influence the outcome of the penalty kick (Almeida et al., 2016). Besides that, the run-up approach angle has shown to be important to predict kick directions (Li et al., 2015). On the contrary high ratings were found to other variables, such as run-up fluency, kicking technique, gaze behavior, goalkeeper performance, the moment of the match, match importance, penalty kick outcome, penalty taker, and goalkeeper strategy. The high ratings confirmed previous findings (Kuhn, 1988; van der Kamp, 2006; Noël and van der Kamp, 2012; White and O’Donoghue, 2013; Li et al., 2015; Noël et al., 2015; Almeida et al., 2016). Studies have shown that the run-up pattern differed between strategies. For penalty takers with the keeper-independent strategy (Kuhn, 1988), the run-up seems to be more fluent, and the total run-up and last step distance is longer than for kicks with the keeper-dependent strategy (Noël et al., 2015). According to Kuhn (1988), ball speed could also distinguish strategies. One characteristic that differentiates the penalty takers’ profiles is the space-temporal pattern of gaze (Noël and van der Kamp, 2012). Those authors found that those penalty takers who use a keeper-dependent strategy spend more time looking at the goalkeeper throughout the run-up and kick execution than penalty takers who use a keeper-independent strategy. The prevalence of penalty kick strategies can also be mediated by personal or situational factors, including a player’s skill or the importance of the kick (Noël et al., 2015).

For intra-rater-reliability, the lowest value found was kappa = 0.75 for the variables non-kicking foot orientation and penalty taker strategy. For inter-rater reliability, the lowest value found was kappa = 0.70 for the variable goalkeeper tactical action. Both values are still good strength of agreement (O’Donoghue, 2010). The low value in the variable non-kicking foot orientation can be explained by the small size of the object of interest compared to the large volume that contains the necessary elements for recording a penalty kick. Although a minimum level of video pixel resolution is required as inclusion criterion for this study, this is not sufficient for precise observations. The variable goalkeeper’s tactical action requires the interpretation of behavior and consequently involves a large amount of subjectivity. Despite having clear definitions in the proposed system, judgments here may be influenced by the sporting experience of each observer or the former playing position (e.g., goalkeeper). The same does hold for the other variables with low kappa values (e.g., strategy assessment). Typically, these variables require a subjective interpretation but are so far only accessible with observational methods. However, the values regarding the level of intra- and inter-observer agreement reached in this study showed that the instrument is reliable when used by trained observers, meeting the minimum thresholds proposed by Altman (1991).

A novelty of this study is the inclusion of viewing angle analysis and video standardization. To the best of our knowledge, there is no other study yet that has included this type of specification for notational studies applied to penalty kick analysis in elite football. The description and standardization of the viewing angles and video quality allow the reproducibility of the instrument and reduce human error. Results indicate that for an optimum penalty kick analysis a combination of at least 3 different viewing angles of the same penalty is recommended. The pitch-level viewing angle behind the penalty taker (d), aerial viewing angle behind the penalty taker (c), and pitch-level viewing angle behind the goalkeeper (e) are the best viewing angles for observation analysis according to a panel of 20 experts in sports science. The change of viewing angles plays an important role in notational analysis, as it may influence the perception of the observer. Depending on the positioning and setting of cameras, recordings may literally provide a different view or perspective of human activity, confirming, complementing, or contrasting what the researchers themselves can see Todd et al. (2007) and LeBaron et al. (2018). Additionally, the standardization of video quality is indicated as a prerequisite for notational analysis. Observational studies in football, specifically in the penalty kick situation using video analysis should describe the pattern of viewing angles presentation, the quantity of angles, and video quality, since these settings may have a direct influence on the perception of the observer. The lack of standardization and indication of this information may compromise the analysis and comparisons of different observational studies. However, it is worth mentioning that the choice of the viewing angles might depend on the research question.

The present methodological design containing a three-study concept made it possible to have a practical approach to the proposed instrument through the pilot study with high-level football coaches. The main study, including three different dimensions (i.e., agreement, adequacy, and univocity) ensured that only variables with a high level of concordance, clarity, and relevance were included in the final format of the instrument. Observational systems, such as the present one, are an important methodology to investigate the structure of sports, guide the coaching and training process, design tactical and technical plans, and develop training methods (Lames and Hansen, 2001; Sarmento et al., 2010; Anguera et al., 2011; Villarejo et al., 2014). Effective observational instruments require high validity and reliability standards, for both the design process and their usefulness for gathering data from competitions (O’Donoghue et al., 2017). A major concern is an extent to which the content of each item of the scale reflects the content domain intended to be measured by the item (Randall et al., 2009). In the present study, based on the judgments of 20 experts, only variables that presented an Aiken’s *V* value above the cut-off value were included in the OSPAF.

The validation of observational variables for penalty shooting may provide a general description of its technical execution, which allows for detecting the shooter’s and the goalkeeper’s strategy based on the behavioral variables studied. These variables, once validated, could be employed to complement and validate subjective strategy judgments. Only a small number of performance analysis studies have examined the validation process of observational instruments applied to penalty kicks (e.g., Noël et al., 2015; Comas et al., 2018). The present study controls content validity and reliability (Aiken’s *V* and observer agreement) through observable and measurable variables, providing a more holistic and contextual analysis rather than the up to now more analytic and reductionist approaches.

Future research with the application of the OSPAF is needed to identify penalty kick strategies and the relationship between the variables in the system itself. One actual key question is the strategy assessment in in-match penalties, as it is necessary to modeling strategies by direct assessment, and further validation of this model by “soft” observational variables contained in OSPAF. A larger representative study in different leagues and female football can contribute to the identification of the successful and failure profile in penalty kicks across different levels. Additional studies can use the OSPAF applying technological methods to analyze its variables, such as gaze analysis by tracking instruments, computer techniques for body pose estimation, and machine learning-based video analysis.

Despite the possible limitation that the study was conducted via an extensive questionnaire, the OSPAF enables the differentiation of the technical and tactical behavior of the goalkeeper and penalty taker. The present instrument is a comprehensive observational system, which contains the most relevant variables for penalty kick analysis validated by experts. The inclusion of observable variables about the penalty taker actions, goalkeeper, context and outcomes, makes the proposed model more complete than most of the others proposed previously. Reliability has been examined per variable; this standard is sometimes not met in other studies, where the assessment is done for the system as a whole (e.g., Noël et al., 2015). In OSPAF, reliability analysis was conducted through the state of the art for validation of observational instruments introducing Aiken’s *V* statistics specially designed for measuring the agreement of several judges. The high methodological rigor of this study consolidates the OSPAF as an instrument that integrates the main variables for penalty analysis in top-level football. Also, evidence for standardization of viewing angles and video quality is presented. Football coaches and match analysts of all levels can use the methodological framework of OSPAF to evaluate and record the penalty kick performance profile of their players throughout the season and using this information for adjusting and improving the coaching process. The final version of the OSPAF is included as a stand-alone supplementary resource, that can be downloaded by researchers and practitioners. For future observational studies of penalties, it is recommended to use OSPAF as a starting point and to add variables specific to the new topic under scrutiny.

## Conclusion

The OSPAF evidenced content validity, inter- and intra-reliability for analyzing penalty kicks in football, through the use of a gold standard methodology for instrument validation. The present study concludes that the final instrument is adequate and consistent for analyzing successful and non-successful penalty kick patterns. Common statistical requirements for the validation of the system presented were met without exception. There are clear operational definitions in the system, and it can be reproduced reliably. The literature gains a validated tool capable of promoting reliable penalty analysis in elite football and provides new guidelines on the standardization of videos in notational systems.

## Data Availability Statement

The raw data supporting the conclusions of this article will be available from the authors upon request.

## Ethics Statement

Written informed consent has not been obtained from the individual(s) for the publication of any images or potentially identifiable data included in this article, as the data is publically available.

## Author Contributions

GP and ML contributed to the conception and design of the study. GP and VN organized the database and performed the statistical analysis. GP, MD, VC, and ML wrote the first draft of the manuscript. GP, ML, and MD wrote sections of the manuscript. All authors contributed to the revision of the manuscript, read, and approved the presented version.

## Conflict of Interest

The authors declare that the research was conducted in the absence of any commercial or financial relationships that could be construed as a potential conflict of interest.
