# Supplementary material for: Design and Validation of an Observational System for Penalty Kick Analysis in Football (OSPAF)
Source: Front Psychol. 2021 May 28;12:661179. doi: 10.3389/fpsyg.2021.661179 (PMC8194359; doi:10.3389/fpsyg.2021.661179)
Supplement: Supplementary file 1 [file Data_Sheet_1.docx]

Supplementary Material

# Supplementary Tables

**Table 1. Aiken’s V values of the observational system for penalty analysis in football (OSPAF)**

|  | Agreement (5-scale) - 95% | | | | Adequacy (5-scale) - 95% | | | | Univocity (2-scale) - 95% | | | |
| --- | --- | --- | --- | --- | --- | --- | --- | --- | --- | --- | --- | --- |
| Variable definition | **M** | ***V*** | **L** | ***V_p_*** | **M** | ***V*** | **L** | ***V_p_*** | **M** | ***V*** | **L** | ***V_p_*** |
| Run up speed | 4.10 | 0.77 | 0.67 | 0.71 | 4.00 | 0.75 | 0.65 | 0.73 | 0.90 | 0.90 | 0.70 | 0.92 |
| Run up fluency | 4.38 | 0.85 | 0.75 | 0.88 | 4.14 | 0.79 | 0.68 | 0.83 | 1 | 1 | 0.84 | 1 |
| Run up approach angle | 3.86 | 0.71 | 0.61 | 0.69 | 3.76 | 0.69 | 0.58 | 0.81 | 0.76 | 0.76 | 0.52 | 1 |
| Number of steps | 3.76 | 0.69 | 0.58 | 0.74 | 3.81 | 0.70 | 0.59 | 0.82 | 0.95 | 0.95 | 0.77 | 0.96 |
| Kicking technique | 4.38 | 0.85 | 0.75 | 0.78 | 4.29 | 0.82 | 0.72 | 0.83 | 1 | 1 | 0.84 | 1 |
| Perceived ball speed | 4.19 | 0.80 | 0.70 | - | 4.10 | 0.77 | 0.67 | - | 0.81 | 0.81 | 0.58 | - |
| Foot used to kick | 3.67 | 0.67 | 0.56 | 0.71 | 3.86 | 0.71 | 0.61 | 0.67 | 1 | 1 | 0.84 | 0.89 |
| Non-kicking foot orientation | 4.24 | 0.81 | 0.71 | 0.86 | 4.00 | 0.75 | 0.65 | 0.82 | 0.90 | 0.90 | 0.70 | 1 |
| Penalty taker gaze behavior | 4.38 | 0.85 | 0.75 | 0.89 | 4.43 | 0.86 | 0.76 | 0.81 | 0.81 | 0.81 | 0.58 | 1 |
| Goalkeeper (GK) initial posture | 3.90 | 0.73 | 0.62 | - | 3.86 | 0.71 | 0.61 | - | 0.95 | 0.95 | 0.77 | - |
| Deception by the penalty taker | 4.19 | 0.80 | 0.70 | 0.84 | 4.24 | 0.81 | 0.71 | 0.83 | 0.86 | 0.86 | 0.64 | 1 |
| Anticipation movement of the goalkeeper at the ball contact point | 4.29 | 0.82 | 0.72 | 0.83 | 4.24 | 0.81 | 0.71 | 0.78 | 0.90 | 0.90 | 0.70 | 0.95 |
| Goalkeeper tactical action | 4.14 | 0.79 | 0.68 | 0.72 | 4.24 | 0.81 | 0.71 | 0.83 | 0.90 | 0.90 | 0.70 | 0.89 |
| Goalkeeper performance | 4.76 | 0.94 | 0.87 | 0.77 | 4.71 | 0.93 | 0.85 | 0.69 | 1 | 1 | 0.84 | 0.90 |
| Moment of the match | 4.48 | 0.87 | 0.78 | 0.84 | 4.14 | 0.79 | 0.68 | 0.75 | 1 | 1 | 0.84 | 1 |
| Location of the match (kicker point of view) | 4.00 | 0.75 | 0.65 | 0.81 | 3.95 | 0.74 | 0.62 | 0.81 | 1 | 1 | 0.84 | 1 |
| Momentary result (kicker point of view) | 4.29 | 0.82 | 0.72 | 0.87 | 4.19 | 0.80 | 0.70 | 0.79 | 1 | 1 | 0.84 | 1 |
| Momentary result (goalkeeper point of view) | 4.24 | 0.81 | 0.71 | 0.83 | 4.10 | 0.77 | 0.67 | 0.80 | 0.95 | 0.95 | 0.77 | 1 |
| Match Importance | 4.38 | 0.85 | 0.75 | - | 4.14 | 0.79 | 0.68 | - | 0.90 | 0.90 | 0.70 | - |
| Penalty kick direction | 4.29 | 0.82 | 0.72 | 0.77 | 4.33 | 0.83 | 0.74 | 0.69 | 0.95 | 0.95 | 0.77 | 1 |
| Penalty kick height | 4.29 | 0.82 | 0.72 | 0.85 | 4.29 | 0.82 | 0.72 | 0.78 | 0.95 | 0.95 | 0.77 | 1 |
| Penalty kick outcome | 4.67 | 0.92 | 0.84 | 0.84 | 4,71 | 0.93 | 0.85 | 0.69 | 1 | 1 | 0.84 | 1 |
| Penalty taker strategy | 4.81 | 0.95 | 0.88 | 0.88 | 4.57 | 0.89 | 0.81 | 0.81 | 0.95 | 0.95 | 0.77 | 1 |
| Goalkeeper strategy | 4.52 | 0.88 | 0.79 | 0.87 | 4.33 | 0.83 | 0.74 | 0.79 | 0.90 | 0.90 | 0.70 | 1 |

**Supplementary Table 1.** Legend: M = median; *V* = Aiken´s *V* value of the main study; L = lower 95% confidence interval limit; *V_p_* = Aiken´s *V* value of the pilot study. Cut-off for 5-scale (p < 0.05) = *V* > 0.64 (n=20); for 2-scale (p<0.05) = *V* > 0.75 (n=20); Agreement: degree of general acceptance of criteria; Adequacy: level of pertinence and importance for criteria and categories specific purpose; Univocity: clarity domain of a definition

**Table 2. Variables, definitions, attribute levels and Aiken’s V values of the excluded variables**

| Variable | Definition | Attribute levels | *V*_AG_ | *V*_AD_ | *V*_U_ |
| --- | --- | --- | --- | --- | --- |
| Run up type | The angle of the penalty kicker's run with the ball | Frontal or diagonal | 0.52 | 0.54 | 0.72 |
| Run up length | How long is the run-up of the penalty kicker | Short run-up or Long run-up | 0.44 | 0.44 | 0.65 |
| Swing behavior of the kicking leg | Kick leg balancing profile of the penalty kicker | Normal or delayed | 0.78 | 0.73 | 0.60 |
| Position of the arm opposite the kicking leg | Position of the penalty kicker's arm during the kick | Low Abduction, Perpendicular to the player's trunk or High Abduction | 0.65 | 0.51 | 0.80 |
| Preparation time | Time after the referee's whistle | > 3 seconds, < 3 seconds | 0.50 | 0.53 | 0.90 |
| Distraction by the goalkeeper | Indication if the goalkeeper has done any action to distract the kicker at any time during the penalty kick | Yes or No | 0.81 | 0.76 | 0.70 |
| Presence of advertisement behind the goal | Indication of if there are advertising boards behind the goal | Yes or No | 0.39 | 0.44 | 1 |

**Supplementary Table 2.** * Results might be interested for studies with thematic focus where these variables play a role. Legend: *V*_AG_ = Aiken´s *V* value of agreement dimension; *V*_AD_ = Aiken´s *V* value of adequacy dimension; *V*_U_ = Aiken´s *V* value of univocity dimension.

**Table 3. Final version of the OSPAF**

| Variables | Definition | Attribute Levels |
| --- | --- | --- |
| Run up speed | Running speed of the penalty kicker towards the ball | Fast or Slow |
| Run up fluency | Characteristic of the penalty kicker's run during the approach of the ball, with or without pauses. | Continuous Running or Running with pauses |
| Run up approach angle | Penalty kicker's running angle to the ball. | Frontal or diagonal |
| Number of steps | Number of steps of the penalty kicker until contact with the ball | 1-3; 3-5 or +5 |
| Kicking technique | The technique used by the penalty kicker to kick the ball | Side foot kick or Instep kick |
| Perceived ball speed | How hard is the ball kicked? | Powerful shot or Placed shot |
| Foot used to kick | Foot used by the penalty kicker to kick the ball | Right or Left |
| Non-kicking foot orientation | Spatial orientation of the penalty kicker's support foot | Same orientation as the final direction of the kick; or Different orientation as the final direction of the kick |
| Penalty taker gaze behavior | Gaze behavior of the kicker during the approach run. | Gaze at the ball or Not at the ball |
| Goalkeeper (GK) initial posture | Position of the body segments. | Arms raised; Arms down or Arms extended in a position perpendicular to the goalkeeper 's trunk |
| Deception by the penalty taker | Indication if the kicker has done any action to distract the goalkeeper during his or her run-up | Yes or No |
| Anticipation movement of the goalkeeper at the ball contact point | Action is performed parallel to the kicker's kick action. | No Movement; Partial movement (at least 1 body segment moved); or Full movement (>1 body segment moved) |
| Goalkeeper tactical action | General evaluation of the way the goalkeeper acted during the penalty shoot-out, to the anticipatory aspect | Try to guess the location of the shot; or Awaiting the penalty taker action |
| Goalkeeper performance | Evaluation of the goalkeeper's performance according to his movement and contact with the ball | 0: GK made any final movement to the side of the goal opposite to the final ball location; 1: GK did not move from the center of the goal; 2: GK made a movement in the correct direction but did not dive and failed to make contact with the ball; 3: GK dived in the correct direction but failed to make contact with the ball; 4: GK dived in the correct direction and contacted the ball without saving it; or 5: GK successfully saved the kick |
| Moment of the match | Time of the match when the penalty will be taken | First half; Second Half or Extra time or Shoot out |
| Location of the match (kicker point of view) | Indication if the penalty kicker is from the home team, visitor, or if he plays on a neutral field. | Home, Neutral or Away |
| Momentary result (kicker point of view) | Result of the match (for the penalty kicker) at the moment the penalty was marked. | Winning, Drawing or Losing |
| Momentary result (GK point of view) | Result of the match (for the Goalkeeper) at the moment the penalty was marked. | Winning, Drawing or Losing |
| Match importance | Level of importance of the match for the team | Championship final match; Decisive knockout match; Group stage match; Early season game; Match in final stages of the season |
| Penalty kick direction | The direction of the ball on goal | Left; Center or Right |
| Penalty kick height | Height of the ball on goal | Upper; Center or Down |
| Penalty kick outcome | Result of the penalty kick | Goal; Saved by goalkeeper or Shot misses goal (wide, over or post) |
| Penalty taker strategy | Overall strategy perceived by the observer (Kuhn, 1988) | Goalkeeper Dependent; Unclear or Goalkeeper independent |
| Goalkeeper strategy | Overall strategy perceived by the observer (Kuhn, 1988) | Kicker Independent; Unclear or Kicker dependent |

# Table 4. Cohen's Kappa for the OSPAF variables.

| Variable | Kappa  Intra-observer | Kappa  Inter-observers |
| --- | --- | --- |
| Run up speed | 0.81 | 0.76 |
| Run up fluency | 1.00 | 0.80 |
| Run up approach angle | 0.85 | 0.80 |
| Number of steps | 0.89 | 0.82 |
| Kicking technique | 0.91 | 0.82 |
| Perceived ball speed | 0.84 | 0.79 |
| Foot used to kick | 1.00 | 1.00 |
| Non kicking foot orientation | 0.75 | 0.81 |
| Penalty taker gaze behavior | 0.78 | 0.78 |
| Goalkeeper initial posture | 0.84 | 0.84 |
| Deception by the penalty taker | 0.92 | 0.81 |
| Anticipation movement of the goalkeeper at the ball contact point | 0.86 | 0.78 |
| Goalkeeper tactical action | 0.77 | 0.70 |
| Goalkeeper performance | 0.86 | 0.83 |
| Moment of the match | 1.00 | 1.00 |
| Location of the match (kicker point of view) | 1.00 | 1.00 |
| Momentary result (kicker point of view) | 1.00 | 1.00 |
| Momentary result (GK point of view) | 1.00 | 1.00 |
| Match Importance | 1.00 | 1.00 |
| Penalty kick direction | 1.00 | 1.00 |
| Penalty kick height | 0.95 | 0.90 |
| Penalty kick outcome | 1.00 | 1.00 |
| Penalty taker strategy | 0.75 | 0.73 |
| Goalkeeper strategy | 0.79 | 0.75 |
| Median value for Kappa | **0.90** | **0.86** |

**Table 5. Aiken’s V for video analysis study on optimum video footage**

| Variable | M | V | L |
| --- | --- | --- | --- |
| Number of angles needed for penalty kick analysis | 0.90 | 0.90 | 0.81 |
| Influence of changing angles on the observer analysis | 0.86 | 0.86 | 0.64 |
| Pre-requisite of video quality | 0.95 | 0.95 | 0.76 |

**Supplementary Table 5.** Legend: M = median; V = Aiken´s V value; L = lower 95% confidence interval limit;. Cut-off for 5-scale (p < 0.05) = V > 0.64 (n=20); for 2-scale (p<0.05) = V > 0.75 (n=20)

## Supplementary Figures


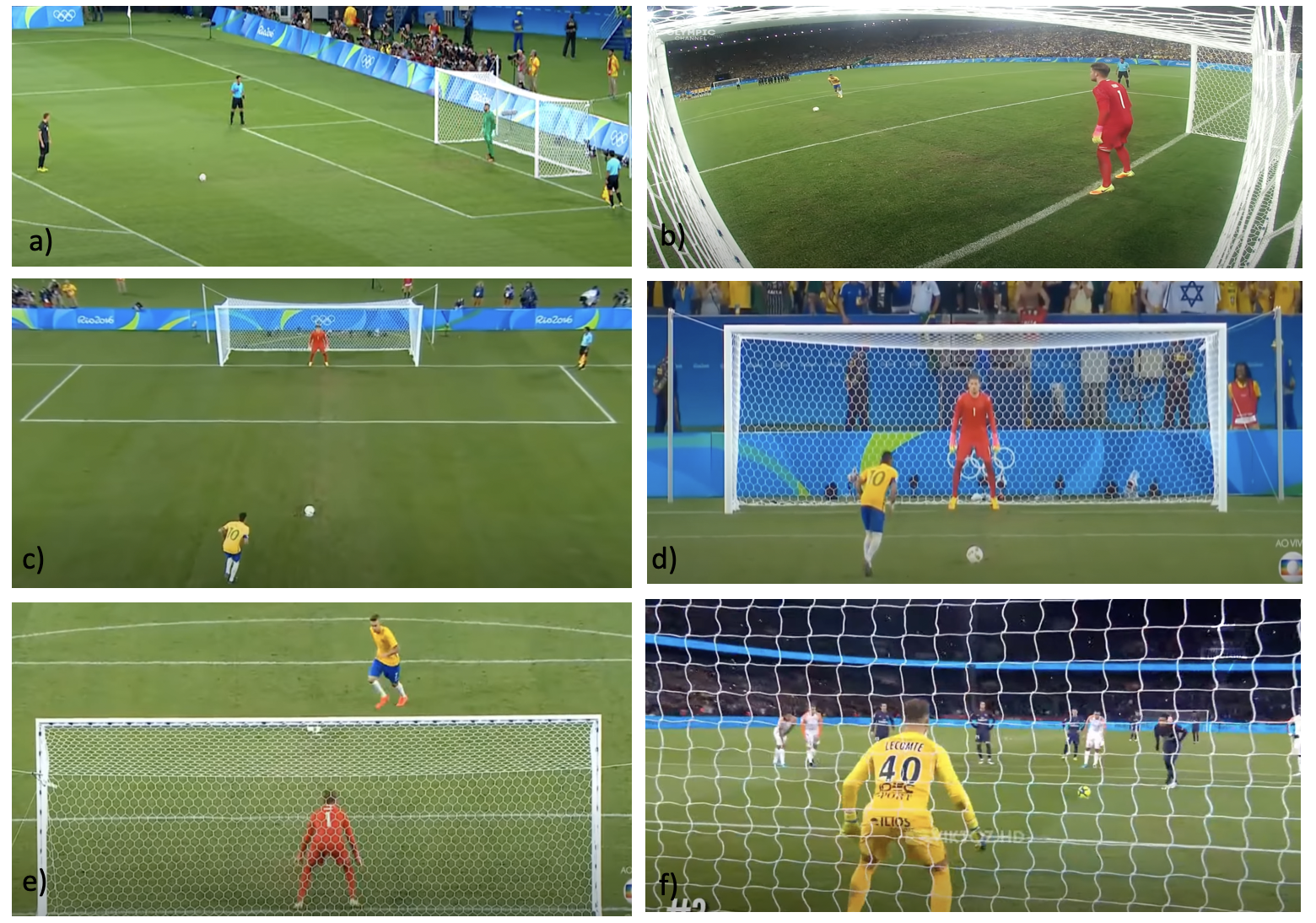


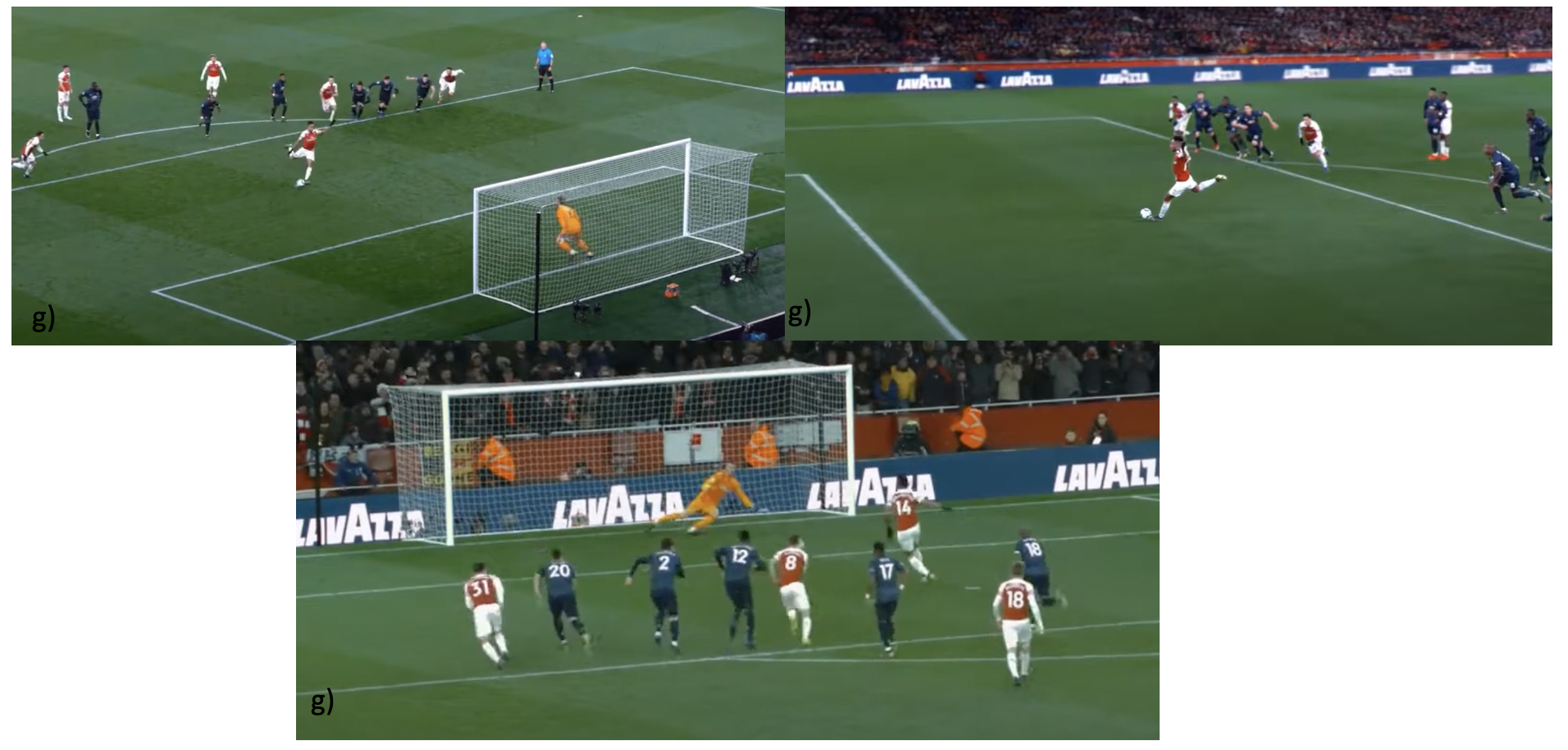


**Supplementary Figure 1.** a) Lateral side; b) Lateral side with camera inside the goal; c) Behind the penalty taker aerial view; d) Behind the penalty taker pitch view; e) Behind the goalkeeper aerial view; f) Behind the goalkeeper pitch view; g) Rotational angle: this viewing is a rotational angle, which occurs when the penalty taker initiates the approach to the ball. The three images show some moments of the rotation. (Figures and videos are of public domain).


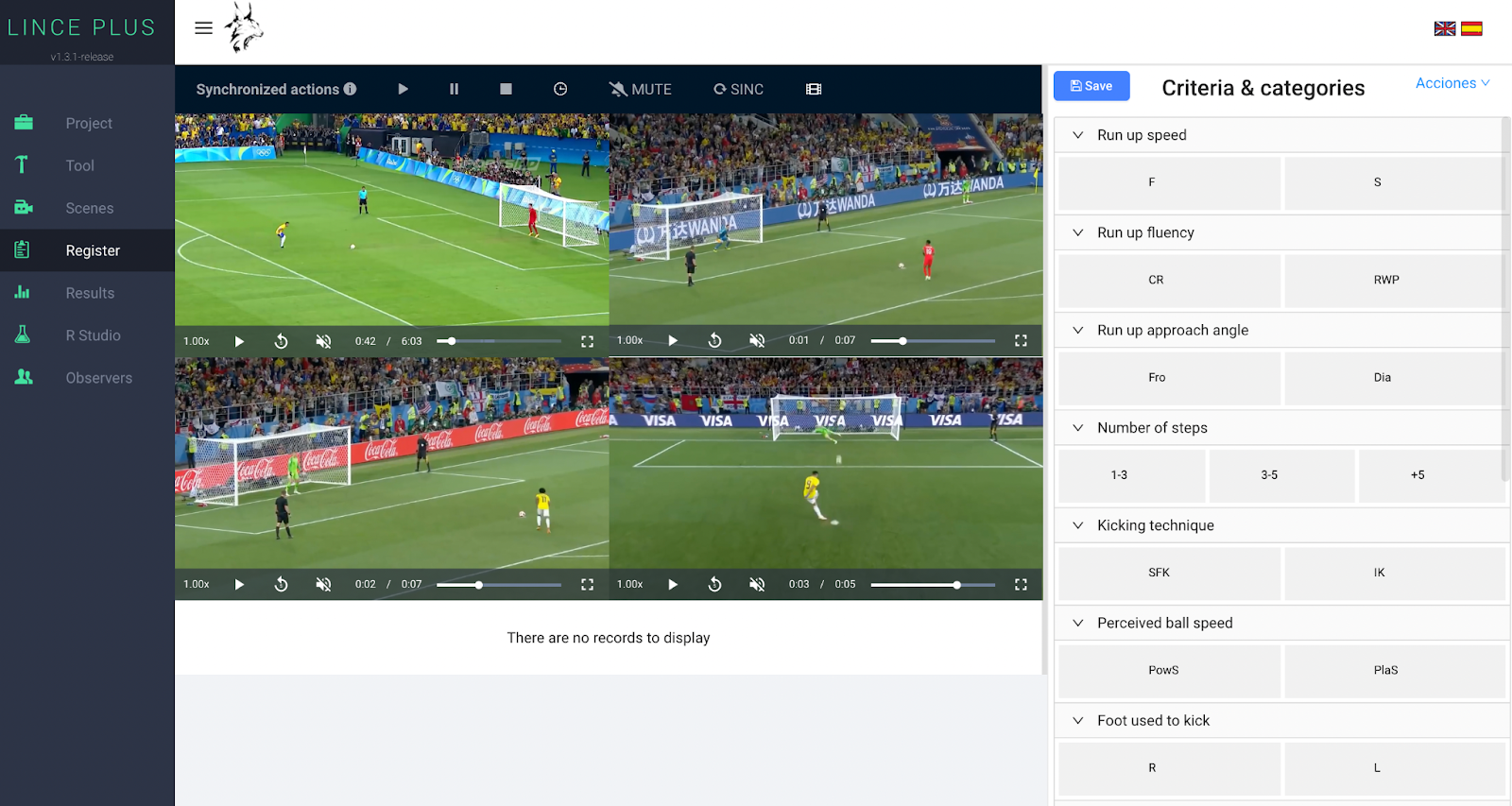
**Supplementary Figure 2.** Lince Plus interface. Criteria and categories example


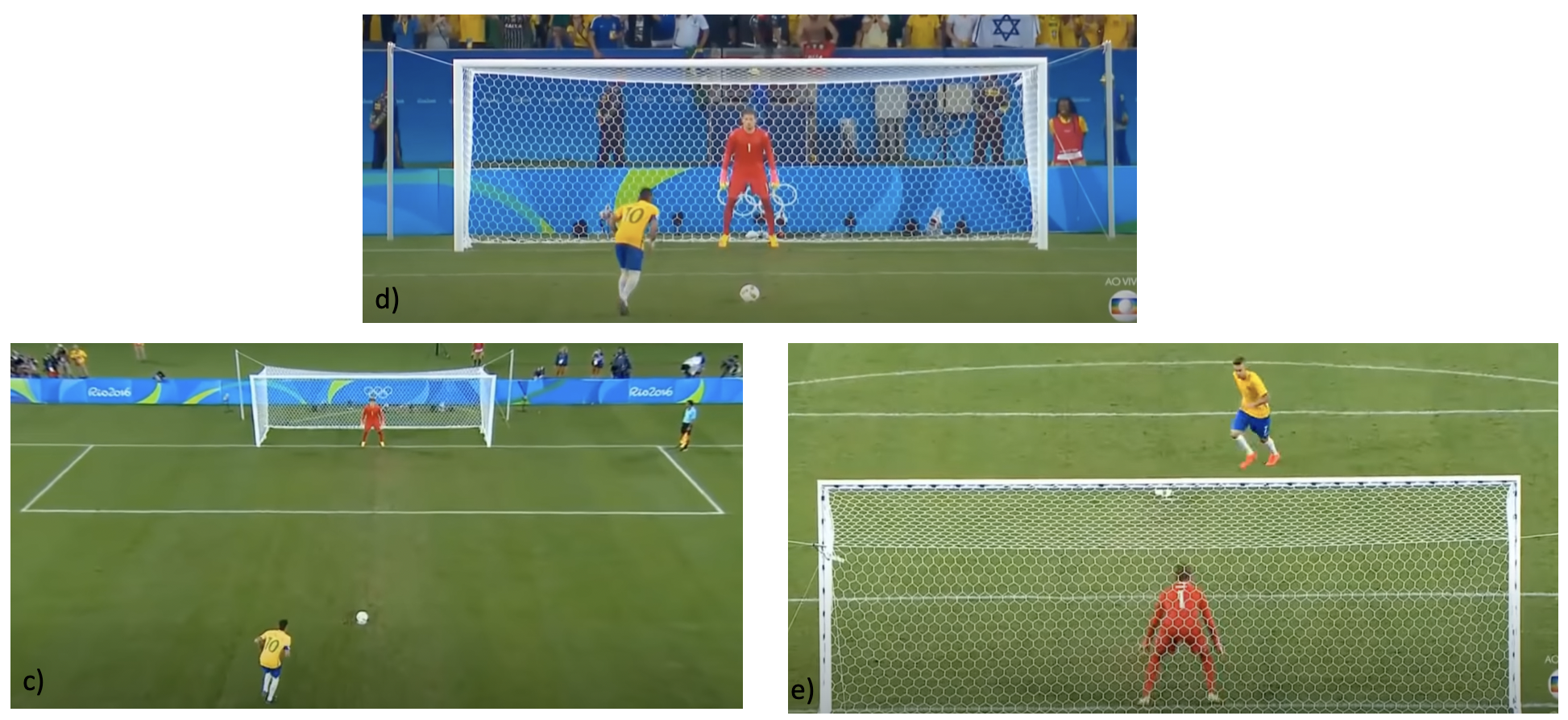


**Supplementary Figure 3.** Optimum viewing angles indicated by experts (Figures and videos are of public domain).
